# Pharmacological effects of Chinese medicine modulating NLRP3 inflammasomes in fatty liver treatment

**DOI:** 10.3389/fphar.2022.967594

**Published:** 2022-09-08

**Authors:** Tingting Liu, Guang Xu, Longxin Liang, Xiaohe Xiao, Yanling Zhao, Zhaofang Bai

**Affiliations:** ^1^ Senior Department of Hepatology, Fifth Medical Center of PLA General Hospital, Beijing, China; ^2^ Military Institute of Chinese Materia, Fifth Medical Center of PLA General Hospital, Beijing, China; ^3^ School of Traditional Chinese Medicine, Capital Medical University, Beijing, China; ^4^ The Third Affiliated Hospital of Zunyi Medical University (The First People’s Hospital of Zunyi), Guizhou, China; ^5^ Department of Pharmacy, The Fifth Medical Center of PLA General Hospital, Beijing, China

**Keywords:** Chinese medicine, NLRP3 inflammasome, inhibitor, NAFLD, ALD, liver fibrosis

## Abstract

Inflammation is a key contributing factor in the pathogenesis of fatty liver diseases (FLD), such as nonalcoholic fatty liver disease (NAFLD) and alcohol-associated liver diseases (ALDs). The NLRP3 inflammasome is widely present in the hepatic parenchymal and non-parenchymal cells, which are assembled and activated by sensing intracellular and extracellular danger signals resulting in the matures of IL-1β/IL-18 and pyroptosis. Moreover, the aberrant activation of the NLRP3 inflammasome is considered the main factor to drives immune outbreaks in relation to hepatic injury, inflammation, steatosis, and fibrosis. Therefore, inhibition of NLRP3 inflammasome may be a promising therapeutic target for FLD. Currently, accumulating evidence has revealed that a number of traditional Chinese medicines (TCM) exert beneficial effects on liver injury via inhibiting the NLRP3 inflammasome activation. Here, we summarized the mechanism of NLRP3 inflammasomes in the progression of FLD, and TCM exerts beneficial effects on FLD via positive modulation of inflammation. We describe that TCM is a promising valuable resource for the prevention and treatment agents against FLD and has the potential to be developed into clinical drugs.

## Introduction

Liver diseases have been a global health concern and ranked as one of the major causes of morbidity and mortality worldwide (Asrani et al., 2019). Among the various forms of liver disease, FLD has become the most common liver disease globally, which is associated with fibrosis and the risk of hepatocellular carcinoma and has been classified into NAFLD and ALD (The global, 2020). The vast majority of Europe and Asia encounter a huge burden of fatty liver pathologies, and about 25% of the European population is affected by NAFLD (Younossi et al., 2018). Moreover, approximately 300 million Chinese people suffer from liver disease. Notably, in contrast to the number of newly HBV-infected patients, the burden of NAFLD and ALD in China continued to grow, paralleling the increase in obesity (Wang et al., 2014; Pimpin et al., 2018; Younossi et al., 2018).

The liver is anatomically and physiologically connected to the gut, leading to the liver being constantly exposed to the gut-derived pathogen-associated molecular patterns (PAMPs), such as microbial and toxins, which trigger immune responses (Zhang et al., 2021a). In addition to PAMPs, hepatic inflammation is also activated by intracellular damage signals, which are released by damaged or dying hepatocytes (called damage-associated molecular patterns, DAMPs) (Kubes and Mehal, 2012). PAMPs and DAMPs can be recognized by pattern recognition receptors (PPRs) to induce PAMPs- and DAMPs-triggered immunity and test studies of PPRs include NOD-like receptors (NLR), Toll-like receptors (TLR), and AIM2-like receptors (AIM2) (Takeuchi and Akira, 2010). NLRP3, as the best characterized NLRs, is activated by PAMPs or DAMPs and promotes the expression of inflammatory cytokines to amplify the inflammatory response. The aberrant activation of NLRP3 inflammasome is considered the main driving force behind excessive immune outbreaks. Increasing studies have indicated that the aberration of the NLRP3 inflammasome is implicated in liver diseases, including drug-induced liver injury (DILI), hepatocellular carcinoma (HCC), cholestatic liver injury (CLI), and autoimmune hepatitis (AIH) (Neumann et al., 2018). For example, previous studies demonstrated that traditional Chinese medicines (TCMs), such as *Epimedii Folium* [*Berberidaceae; Epimedium brevicornu Maxim.*], *Psoraleae Fructus* [*Leguminosae; Psoralea corylifolia L.*]*,* and *Sophora flavescens [Leguminosae; Sophorae flavescentis Radix]*, as well as some chemical drugs carbamazepine, isoniazid, and nevirapine, promote NLRP3 inflammasome activation and result in liver injury (Wang et al., 2019a; Gao et al., 2021; Qin et al., 2021; and Lin et al., 2022). A clinical study showed that NLRP3 inflammasome activation exhibits a protective effect on the development of HCC, but other experimental data indicated that NLRP3 deficiency in HCC cells enhance surveillance of NK cells to delay the tumor development in the xenograft mice model (Wei et al., 2014; Lee et al., 2021). Some research studies showed a high level of NLPR3 expression in cholestatic liver injury via the S1P/S1PR2 pathway (Hou et al., 2021). Over-activation of the NLRP3 inflammasome has also been found in trichloroethene- and ConA-induced autoimmune hepatitis mice models, indicating that the inflammasome activation-dependent IL-1β and pyroptosis contributed to exacerbating the liver injury (Luan et al., 2018; Wang et al., 2019b). Emerging evidence revealed that NRLP3 inflammasome activation is a driver of the pathological process of FLD (including NAFLD and ALD), which contributes to hepatic steatosis, liver tissue damage, and necrotic cell death (Del Campo et al., 2018). In both NASH and ALD patients, the level of IL-1β was increased and contributed to the progression of the disease (Tilg et al., 1992; Henao-Mejia et al., 2012). In accordance with human NAFLD and ALD patients, the expressions of NLRP3 and IL-1β were significantly increased in NAFLD and ALD mouse models (Knorr et al., 2020). Hence, NLRP3 inflammasomes might be a novel target for the treatment of liver disease, especially in FLD.

Currently, there is still no availability of approved pharmacological agents approved for the management of ALD and lifestyle modification, such as weight loss and alcohol abstinence, is considered the best therapeutic strategy (Pessione et al., 2003; Ferro et al., 2020). Several market-available drugs have been evaluated in ALDs, such as vitamin E, metformin, and statins, but most of them only provide limited success (Sanyal et al., 2010; Ford et al., 2015; Tziomalos et al., 2015). Thus, there is an urgent need to identify a high efficacy and minimal side effects treatment for ALD. Traditional Chinese medicine (TCM) has a long history of complementary and therapy applications in many countries (Zhang et al., 2021b), and the efficacy and safety for many diseases have been widely verified via long-term empirical trials (Li et al., 2017; Yang et al., 2019). Recently, TCM has gained much attention as a potential application in the prevention and treatment of FLD due to the characteristic of multi-targets, multi-pathway, and less toxic side effects. ALDs are referred to as “Gan-Pi” (NAFLD) or “Jiu-Pi” (ALD), respectively, due to the different etiology in Chinese medicine, and “internal retention of phlegm and dampness”, “liver qi stagnation”, “blood stasis”, and “a deficiency of spleen or kidney” is considered as its pathogenesis (Zhu et al., 2021a; Dong et al., 2012; 张欢 et al., 2022). Thus, the main principle of Chinese medicine in the treatment of ALD involves evacuating phlegm and dampness from the body, relieving qi stagnancy in the liver, removing blood stasis, and strengthening the function of the spleen and kidney (Dong et al., 2012; 张欢 et al., 2022). According to the therapeutics in Chinese medicine, numerous Chinese herbal formulations have been proposed and used for FLD (Tables 1, 2).

**TABLE 1:** Therapeutic effects of traditional Chinese medicine formulas on FLD.

Chinese medicine formulas	Common composition	Model	Effect	Mechanisms	Ref
Gegen Qinlian decoction	*Pueraria lobata (Willd.) Ohwi* [Leguminosae; *Pueraria Lobata Radix*]*, Coptis chinensis Franch* [Ranunculaceae; *Coptidis Rhizoma*]*, Scutellaria baicalensis Georgi* [Lamiaceae; *Scutellariae Radix*]*, Glycyrrhiza*	HFD fed rat model	Decrease serum triglyceride, cholesterol, total bile acid, low-density lipoprotein, free fatty acid, and LPS level	Inhibiting TLR4 signal pathways	Zhang et al. (2020)
	(8:3:3; 2)				
Fuzi Lizhong decoction	*Codonopsis tangshen Oliv* [Campanulaceae; *Codonopsis Radix*]*, Zingiber officinale Rosc.* [Zingiberaceae; *Zingiberis Rhizoma*]*, Aconitum carmichaelii Debx* [Ranunculaceae; *Aconiti Radix Cocta*]*, Glycyrrhiza, Baizhu;* (15:9:6:9:9)	HFD-fed rat model	Reduce serum total cholesterol, triglyceride, blood glucose, and fatty acid in the liver	Activating p53 and inhibiting PPARG signaling	Yang et al. (2020)
Lanzhang granules	*Gynostemma pentaphyllum (Thunb.) Makino* [Cucurbitaceae; Gynostemma]*, Astragalus membranaceus*(*Fisch.*)*Bge.* [Leguminosae; *Astragali Radix*]*, Ephedra sinica Stapf* [Ephedraceae; *Ephedrae Radix Et Rhizoma*]*, Fritillaria pallidiflora Schrenk* [Liliaceae; *fritillariae Pallidiflorae Bulbus*] (30:30:15:20:9)	HFD-fed mice model	Improve lipid metabolism and inflammation, decrease serum ALT and AST levels	Regulation of the PPARα signaling pathway	Huang et al. (2022)
Yiqihuoxue formula	*Gardenia jasminoides Ellis* [Rubiaceae; *Cardeniae Fructus*]*, Rhodiola crenulata* (*Hook. f. et Thoms.*)*H. Ohba* [Crassulaceae; *Rhodiolae Crenulatae Radix Et Rhizoma*]*, Curcuma Longa L.* [Zingiberaceae; *Curcumae Longae Rhizoma*]*, Ligustrum lucidum Ait.* [Oleaceae; *Ligustri Lucidi Fructus*] (1:1:1:1)	HFD-fed rat model	Decrease serum ALT level and hepatic fatty deposition, upregulate serum gastrin and motilin	_	Chen et al. (2013)
Lingguizhugan decoction	*Poria, Cinnamomum cassia Presl* [Lauraceae; *Cinnamomi Ramulus*]*, Baizhu, Glycyrrhiza* (12:9:9:6)	HFD-fed rat model	Alleviate hepatic steatosis and reduce N6-methyladenosine level	N6-methyladenosine modification-mediated suppressor of cytokine signaling	Dang et al. (2020)
Qianggan formula	*Artemisia capillaris Thunb.* [Asteraceae; *Artemisiae Scopariae Herba*]*, Isatis indigotica Fort.* [Brassicaceae; *Isatidis Radix*]*, Angelica sinensis*(*Oliv.*)*Diels* [Apiaceae; *Angeicae Sinensis Radix*]*, Paeonia lactiflora Pall.* [Ranunculaceae; *Paeoniae Radix Alba*]*, Danshen, Curcumae Radix, Curcuma wenyujin Y.H.ChenetC.Ling* [Zingiberaceae, *Astragali Radix, Codonopsis Radix*]*, Zexie, Polygonatum sibiricum Red.* [Liliaceae, *Polygonati Rhizoma*]*, Dioscorea opposite Thumb.* [Dioscoreaceae; *Dioscoreae Rhizoma, Crataegi Fructus*]*, Medicated Leaven Massa Medicata Fermentata, Gentiana macrophylla Pall.* [Gentianaceae; *Gentianae Macrophyllae Radix*]*, Glycyrrhizae*; (10:5:5:5:10:5:10:5:5:5:5:5:4:4:4:4)	MCD-fed mice model	Alleviated liver inflammation, TNF-α, IL-β expression, reduce serum ALT and AST levels	Regulate gut microbiota-mediated LCA production, promote TGR5 expression, and suppress the NF-ƙB activation	Li et al. (2020)

**TABLE 2:** Therapeutic effects of Botanical drugs and natural products on FLD.

Type	Botanical drug/natural product	Model	Effect	Mechanisms	Ref
Extracts	Powder of *Platycodon grandiflorus* [Campanulaceae; Platycodonis Radix]	HFD-fed mice model	Improve hyperlipidemia, liver steatosis, oxidative stress, inflammation, and insulin resistance	Activate the PI3K/Akt/GSK3 pathway	Ke et al. (2020)
	Ethanol extracts from Coix lacryma-jobi L. [Poaceae; *Coicis Semen*]	HFD-fed mice model	Alleviated liver steatosis and inflammation	Inhibit liver lipogenesis and induce fatty acid β-oxidation	Chiang et al. (2020)
	Water extracts from Coix lacryma-jobi L				
	Ethanol extracts from *Cassia obtusifolia L.* [Leguminosae; C*assiae Seme*n]	HFD-fed mice model	Alleviate lipid accumulation, intestinal barrier damage, liver injury, and hepatic inflammation	Regulate gut microbiota	Luo et al. (2021)
	Ethanol extracts from *Morus alba L.* [Moraceae; *Mulberry leaves*]	Alcohol-fed mice model	Decrease cyclooxygenase-2, TNF-α, and IL-6 expression, improve hepatocyte apoptosis	Anti-oxidative	Liang et al. (2021)
	Ethanol extracts from Portulaca oleracea L. [Portulacaceae; Po*rtulacae Herba*]	Alcohol-fed rat model	Decrease serum ALT, AST, ALP, triglyceride levels, hepatic NO, MDA, TNF-α, and IL-6 level	Regulate lipid metabolism	Qiao et al. (2019)
	Polysaccharide of *Schisandra chinensis*(*Turcz.*)*Baill.* [Magnoliaceae; *Schisandr*ae Chinensis Fructus]	HFD-fed mice model	Decrease serum triglycerides, total, alleviate hepatocyte fatty degeneration and necrosis	Downregulate LXRα/SREBP-1c/FAS/ASC and SREBP-2/HMGCR signaling pathways	Wang et al. (2016)
	Patchouli Oil [Lamiaceae; *Pogostemon cablin(Blanco) Benth..* ]	HFD-fed rat model	Decrease lipid profiles and serum enzymes	Decrease *de novo* lipogenesis, promote export of lipids, improve fatty acids oxidation	Xu et al. (2020)
Flavonoids	Isoquercetin	HFD-fed rat model	Improve liver lipid accumulation, inflammation, and oxidative stress	Activate the AMPK pathway and suppress the TGF-β signal	Qin et al. (2018)
Adenosines	Cordycepin	HFD-fed mice model	Decrease serum aminotransferases, hepatic triglyceride, inflammation, and fibrosis	Activate the AMPK signaling pathway	Lan et al. (2021)
Phenols	Curcumin	High-fat- and high-fructose- fed mice model	Improve hepatic steatosis and serum biochemical parameters	Regulate the Nrf2/FXR/LXRα pathway	Yan et al. (2018b)
		Alcohol-fed mice model	Improve hepatocyte necroptosis	Regulat4e Nrf2/p53 pathway	Lu et al. (2016)
Phenols	Gastrodin	Alcohol-fed mice model	Reduce serum ALT, AST, and MDA levels, hepatic glutathione peroxidase, and catalase expression	Enhance Nrf2 translocation to the nucleus	Li et al. (2019)

Emerging immunological studies also show that NLRP3 inflammasomes play an important role in the development of FLD and might be a promising therapeutic target for the treatment of FLD. Moreover, a variety of Chinese herbal formulations, TCM extracts, and natural products exert a wide range of anti-inflammatory effects by inhibiting the activation of the NLRP3 inflammasome and showing a potent and effective effect in various FLD (Fan et al., 2020; Wang et al., 2022). In this review, we systematically summarized the role and mechanisms of NLRP3 inflammasome activation in FLD and how the TCM targets and regulates NLRP3 inflammasome to improve the development of FLD.

### The activation of NLRP3 inflammasomes and potential modulating factors

**FIGURE 1:**
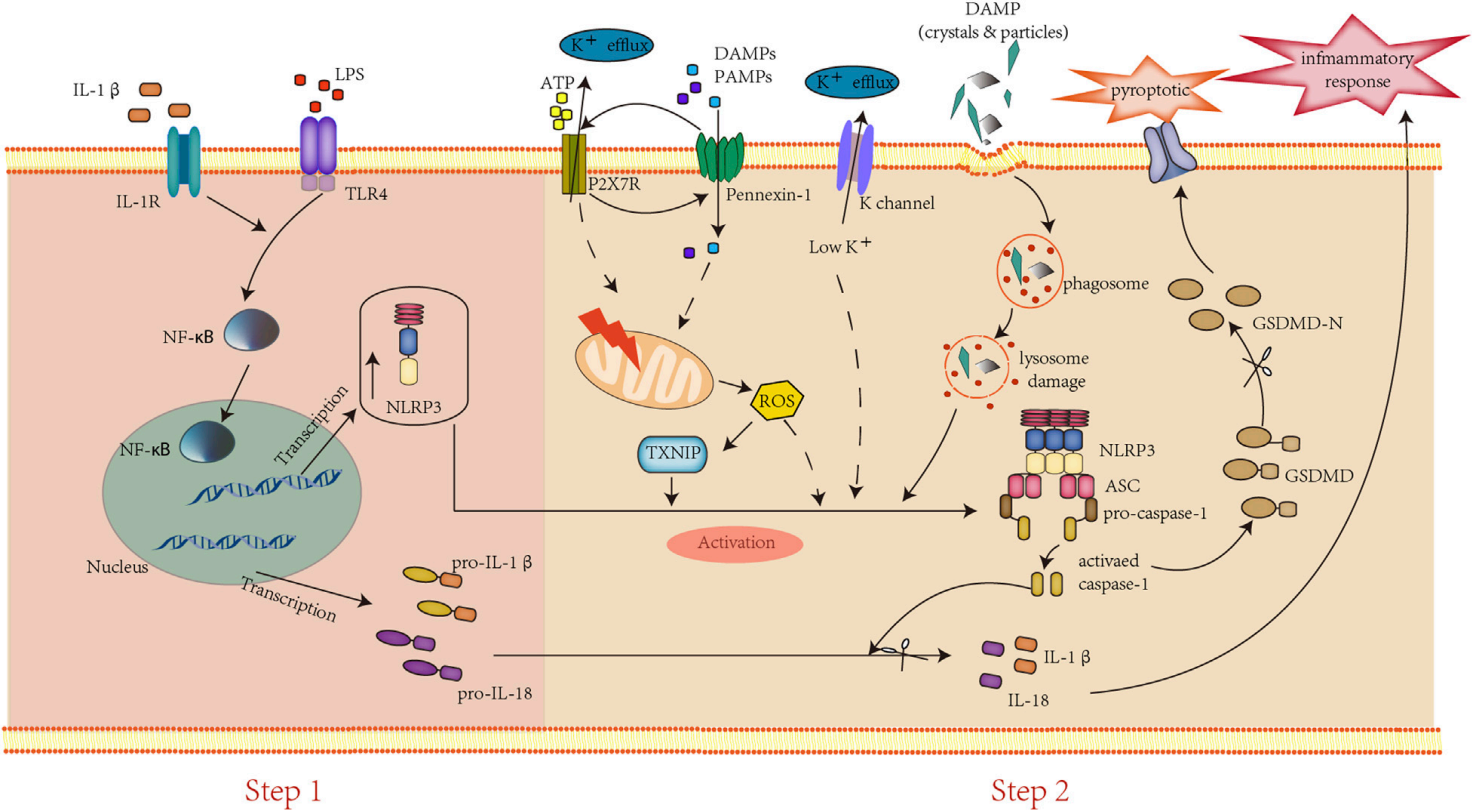
Assemble and activation of the NLRP3 inflammasome.

NLRP3 inflammasomes are well known as cytosolic multiprotein complexes consisting of the innate immune sensor protein NLRP3 (also called Cryopyrin), adaptor speck-like protein (ASC), and the caspase-1 protease (Deng et al., 2019). Some studies indicated that NLRP3 may act as a sensor of the homeostatic intracellular process that is activated by sensing the intracellular and extracellular PAMPs and DAMPs (Masters et al., 2010). Typically, the NLRP3 inflammasome activation requires a two-step process, including priming and activating (Figure 1). First, the priming step is usually induced by lipopolysaccharide (LPS), activating the transcription factor nuclear factor-kappa B (NF-ƙB) to upregulate the transcription of inflammasome proteins and pro-cytokines (pro-IL 1β, pro-IL-18). Second, the activating step is provided by a diverse group of DAMPs and PAMPs, such as ATP, cholesterol, reactive oxygen species (ROS), etc., that assemble and activate the NLRP3 inflammasome through three main pathways. 1) Extracellular ATP binds to the ionotropic P2X purinoceptor7 (P2X7) and activates the NLRP3 inflammasome by inducing intracellular K^+^ efflux (Carta et al., 2006). Moreover, the persistently activated P2X7 recruit membrane pore protein pannexin-1 and presumably formed, the “P2X7-PANX1 pore complex”, which allows a variety of PAMPs and DAMPs into the cytosol to trigger NLPR3 inflammasome activation (Kanneganti et al., 2007). 2) The endocytosis of crystals or large particles (amyloid, silica, cholesterol, etc.) induced lysosomal damage, leading to their components and lysosomal proteases release to induce NLRP3 inflammasome activation (Halle et al., 2008; Hornung et al., 2008; Broz and Dixit, 2016). 3) The increase of ROS leads to thioredoxin-interacting protein (TXNIP) translocating from the nucleus to the cytoplasm and bound to thioredoxin to associated with NLRP3 inflammasome activation (Brocker et al., 2020). Subsequently, the activated NLRP3/ASC/pro-caspase-1 complex converts pro-caspase-1 to caspase-1, which in turn processes the mature pro-IL-1β/pro-IL-18 into their secretory bio-active (IL-1β/IL-18) forms, triggering the inflammatory cascade and gasdermin D (GSDMD) cleavage (Basiorka et al., 2016). In addition to the above three main pathways, numerous NLRP3-interacting proteins, including mitosis A-related kinase-7 (NEK7), heat shock protein 90 (HSP90), etc., have been proved to promote the activation of NLRP3 inflammasomes (Duan et al., 2020).

Historically, inflammasomes are central to regulating liver diseases, which has been attributed to their ability to induce hepatic inflammation by up-regulating the expression of IL-1β/IL-18. Increasing clinical and experimental studies have demonstrated that inflammasome activation-dependent IL-1β is a major cause and contributes to liver disease progression (Iracheta-Vellve et al., 2017). The secreted active IL-1β synergistic action with TLR signaling amplifies inflammation by increasing the expression of pro- IL-1β, TNF, CCL2, etc. (Granowitz et al., 1992; Mandrekar et al., 2011). Moreover, IL-1β promotes hepatic stellate cells (HSCs) activation, resulting in liver fibrosis, as well as enhances the accumulation of triglyceride and hepatocyte injury contributing to liver steatosis (Miura et al., 2010; Petrasek et al., 2011). Compared to IL-1β, IL-18 aggravates NASH severity *via* altering the gut microbiota, and it has been proved in the MCD diet-induced NASH model that IL-18-deficient mice progressed to severe NASH more than the control group (Henao-Mejia et al., 2012). However, the role of IL-1β/IL-18 in NAFLD and ALD remains to be elucidated, and the underlying mechanisms require deep investigation.

The activation of the NLRP3 inflammasome requires two steps: first, DAMPs, PAMPs, or cytokines bind to their receptors, leading to the NF-κB signaling pathway activation, resulting in the increased expression of pro-IL-1β/IL-18 and inflammasome components. Second, several DAMPs and PAMPs induce the activation of the NLRP3 inflammasome to trigger caspase-1 cleavage and IL-1β/IL-18 mature. Subsequently, activated caspase-1 cleaves GSDMD to GSDMD-N, resulting in pyroptosis.

### TCM for the treatment of NAFLD by inhibiting the NLRP3 inflammasome

NAFLD is the most frequent type of FLD, affecting more than 20% of people worldwide, and is highly correlated with obesity and metabolic syndrome (Lee et al., 2020). The clinical spectrum of NAFLD is spammed from noninflammatory isolated hepatic steatosis, NASH, progressive to cirrhosis, or even carcinoma (HCC) (Arab et al., 2018). NASH is a severe liver condition that is characterized by hepatocellular damage, steatosis, inflammation, and fibrogenesis. Approximately 10–30% of patients with NAFLD will develop NASH (Liang et al., 2018a). A “two-hit” hypothesis that explains the progression of NAFLD into NASH. The “first hit” involves an abnormal accumulation of lipid and insulin resistance that leads to hepatic steatosis, thereby resulting in the liver being susceptible to “second hits” including dysfunction of mitochondria, endotoxins, inflammation, and oxidative stress. Emerging evidence has indicated that NLRP3 inflammasome activation is implicated in metabolic syndrome, obesity, and NAFLD (Szabo and Csak, 2012; Lee et al., 2013; Esser et al., 2014). Recently, increasing clinical and experimental studies have shown that the expression of NLRP3 inflammasome components (NLRP3, caspase-1, and ASC) was remarkably increased in the patients with NAFLD and in the mice model (Wree et al., 2014; Mitsuyoshi et al., 2017; Gaul et al., 2021). Moreover, both NLRP3 inflammasome components, deficient or treated with NLRP3 inhibitors, attenuated the inflammation, liver fibrosis, and liver cell death in a mouse model, which further demonstrated the role of the NLRP3 inflammasome in NAFLD (Dixon et al., 2013; Li et al., 2022). In animal models, feeding rodents a diet deficient in methionine and choline (MCD) is a classic method of inducing NASH, as well as a prolonged high-fat diet (HFD) and high-fat/high-cholesterol/high-sugar diet (HF-HC-HSD). It is noteworthy that the short period of HFD or HF-HC-HSD feeding causes hepatic steatosis but not NASH (Ganz et al., 2015; Farrell et al., 2019).

Most types of TCMs, including TCM formulas, extracts, and its natural products, have been used in treating NAFLD and exhibit a promising treatment efficacy *via* modulating a variety of risk signals in the process of NAFLD, such as oxidized lipids, DAMPs, and ROS, resulting result in NLRP3 expression in liver tissue through TLR4 (Farrell et al., 2018; Wang et al., 2020; Zhang et al., 2020). Many of them showed a potent effect in modulating NLRP3 inflammasome activation by regulating inflammasome activation-associated signaling pathways, such as the release of ROS, LPS, NF-κB, toll-like, etc. Dansheng Zexie decoction is the water extract of three Chinese medicines, including Salvia miltiorrhiza Bunge (Danshen, 15 g) [ Lamiaceae; Salviae miltiorrhizae radix and rhizoma], *Alisma plantago-aquatica Linn.* (Zexie, 30 g) [Alismataceae; *Alismatis Rhizoma*], and *Atractylodes macrocephala Koidz.* (Baizhu, 12 g) [Asteraceae, *Atractylodis Macrocephalae Rhizoma*]. Both single compound and formulae of the Dansheng Zexie decoction have the effect of modulating cholesterol metabolism to decrease the level of lipid, showing a significant ability for treatment for NAFLD (Ding et al., 2019; Wu et al., 2021; Cao et al., 2022). Recently, experimental research revealed the mechanism underlying Dansheng Zexie decoction in treating NAFLD, which reduces lipid accumulation and alleviates hepatic steatosis, oxidative stress, and inflammation via inhibiting the ROS/NLRP3/IL-1β signaling pathway (Biao et al., 2022). Shenling Baizhu powder is composed of *Dolichos lablab L.* (4 g) [Leguminosae; *Lablab Semen Album*], *Poria cocos (Schw.) Wolf* (5 g) [Polyporaceae; Poria], *Glycyrrhiza* (3 g) [Leguminosae, *Glycyrrhizae Radix Et Rhizoma*], *Platycodon grandiflorus* (2 g) [Campanulaceae; Platycodonis Radix], *Nelumbo nucifera* (3 g) [Nymphaeaceae; *Nelumbinis Semen*], *Panax ginseng C. A. Mey.* (PG, 5 g) [Araliaceae, *Ginseng Radix Et Rhizoma*], *Amomum villosum Lour.* (2 g) [Zingiberaceae; *Amomi Fructus*], *Dioscorea opposita Thunb.* (5 g) [Dioscoreaceae; *Dioscoreae Rhizoma*], *Coix lacryma-jobi L. var.ma-yuen (Roman.) Stapf* (3 g) [Poaceae; Coicis Semen], and Baizhu (5 g), which has been proven to reduce body weight, serum-free fatty acid, and ameliorated liver microcirculation and ultrastructural abnormalities via inhibiting the TLR4/NLRP3 signaling pathway in the HFD-fed rat model (Pan et al., 2021). Chaihu–Shugan–San (CSS) decoction is composed of seven kinds of botanical drugs, including *Citrus reticulata Blanco* (6 g) [Rutaceae; *Citri Reticulatae Pericarpium*], *Bupleurum chinense DC.* (6 g) [Apiaceae; *Bupleuri Radix*], *Ligusticum chuanxiong Hort.* (5 g) [Apiaceae, *Chuangxiong Rhizoma*], *Cyperus rotundus* (5 g) [Cyperaceae; *Cyperi Rhizoma*], *Citrus aurantium* (5 g) [Rutaceae; *Aurantii Fructus*], *Paeonia lactiflora* (5 g) [Ranunculaceae; *Paeoniae Radix Alba*], and *Glycyrrhiza* (3 g)*,* and has been frequently used for the prevention and treatment of chronic diseases (NAFLD and gastroenteropathy), significantly improving lipid peroxidation, inflammation, and liver fibrosis (Yang et al., 2014). The CSS contributes to the reducing serum LPS level, NLRP3 expression, liver steatosis, and reconstruction of the intestinal microflora in the HFD-fed rat model, all processes associated with the NLRP3 inflammasome pathway, suggesting that the inhibition of NLRP3 inflammasome activation is responsible for the treatment of NAFLD with CSS (Liang et al., 2018b).

*Antrodia cinnamomea* (AC) [Polyporaceae; *Antrodia camphorata*], a fungus of the Fomitopsidaceae family, has been used for treating many kinds of diseases and showed an effect in reducing hepatic triglycerides and total cholesterol concentrations in the HFD hamster model. Recent studies demonstrated that the AC ethanol extract attenuated steatohepatitis, oxidative stress, hepatic inflammation, ameliorating the MCD-diet-induced NAFLD by inhibiting NLRP3 inflammasome activation (Yen et al., 2020). Honey, a natural substance produced by bees from nectar, is a classical medicinal and edible TCM that has been investigated and used for various diseases, such as chemical-induced liver injury, hepatic cancer, and diabetes (Erejuwa et al., 2010; El‐kott et al., 2012; Al-Yahya et al., 2013). In recent studies, honey has been used for treating NAFLD and showed a potent effect in improving hepatic histology, lipid metabolism, oxidative stress, and hepatic inflammation via inhibiting the TXNIP-NLRP3 pathway in the HFD-fed rat model (Xiao et al., 2016). *Rheum palmatun L.* (RP) [Polygonaceae; Rhei Radix Et Rhizoma] is one of the most used TCM for “pursing fire and detoxification” and “promoting blood circulation for removing blood stasis” in clinical Chinese medicine, which is frequently prescribed for treating a set of metabolic disorders, and the RP aqueous extract has been reported to ameliorate NAFLD (Yang et al., 2016). Recent studies demonstrated that the RP aqueous extract improved the MCD diet-induced serum inflammation and liver function by inhibiting the activation of NLRP3 inflammasome *in vivo* (Wu et al., 2022).

In addition, numerous natural products isolated from TCMs have been shown to address the treatment potentials of NAFLD through modulating the NLRP3 inflammasome. Increasing studies showed that many natural products of GL exhibit a potent effect in the treatment of *NAFLD. Glycyrrhiza uralensis Fisch.* (GL) is one of the most popular TCM in clinical Chinese medicine shows a wide range of biological activities and common therapies for multisystem inflammatory diseases, such as NAFLD (El-Saber Batiha et al., 2020). A randomized double-blind clinical trial of treating NAFLD demonstrated that licorice, the powder from the root of GL, supplementation contributes to a reduction of ALT levels and liver steatosis in patients with lifestyle modification, suggesting that licorice supplementation can improve the effectiveness of lifestyle modification alone in treating NAFLD (Rostamizadeh et al., 2022). Our group’s previous studies showed that licochalcone B (flavonoids from *GL.*) inhibits NLRP3 inflammasome activation by preventing NEK7 from binding to NLRP3, and echinatin (flavonoids from *GL*) showed a negative effect on NLRP3 inflammasome activation by binding to HSP90. Furthermore, both licochalcone B and echinatin attenuate the MCD-induced increase of alanine transaminase (ALT) and aminotransferase (AST), liver inflammation changes, hepatic steatosis, and fibrosis (Xu et al., 2021; Li et al., 2022). In addition, another study also found that both glycyrrhizin and glycyrrhetinic acid (terpenoids from GL) alleviated the degree of inflammation infiltration and lipid disruption in MCD-diet mice (Yan et al., 2018a). Baicalin (a flavonoid glycoside from *Scutellaria baicalensis Georgi* [Lamiaceae; Scutellariae Radix] significantly reduced NLRP3, gasdermin D (GSDMD), IL-1β expression, and protected hepatocytes from free fatty acids-induced morphological damage and death, protecting hepatocytes from apoptosis by blocking NLRP3-GSDMD signaling *in vitro* (Shi et al., 2020a). Berberine is an isoquinoline alkaloid isolated from numerous herbal plants, which significantly ameliorated lipid accumulation, reducing TNF-α expression and phosphorylation of NF-κB, and inhibiting NLRP3 inflammasome activation by modulating the ROS/TXNIP axis in MCD-diet mice model (Mai et al., 2020). Emodin, rhein, diacerein, aloe-emodin, and 1,8-dihydroxyanthraquinone are free anthraquinones from *Rheum palmatum L.*, and all of them remarkably decreased serum ALT, AST, IL-1β, and TNF-α levels, improved hepatic inflammation, and fibrosis by blocking the activation of the NLRP3 inflammasome and the underlying mechanism of this role is related to inhibited ASC oligomerization.

### TCM for the treatment of ALD by inhibiting the NLRP3 inflammasome

A major cause of ALD is due to the intake of excessive alcohol and is similar to NAFLD in pathology, ranging from hepatic steatohepatitis to fibrosis and cirrhosis (Silva et al., 2017). The early stage of ALD can be reversible with limited alcohol intake, but the advanced stages (including cirrhosis and severe alcoholic hepatitis) are irreversible, with fatal outcomes mediated by liver failure (Szabo et al., 2006). Activation of NLRP3 inflammation plays an important role in the progression of ALD, and the increased IL-1β and neutrophilia are characteristic features of sterile inflammation. The expression of IL-1β is significantly increased in patients with ALD, which is more than 10 times higher than in healthy controls (McClain et al., 1986). Moreover, the level of NLRP3 inflammasome components and IL-1β are increased in mice fed with excess ethanol (Petrasek et al., 2012). Similarly, caspase-1 or ASC deficient and IL-1 receptor knockout mice showed decreased ethanol-induced hepatic injury and steatosis (Petrasek et al., 2012). Moreover, liver macrophages (Kupffer cells) were important in mediating inflammasome activation in the progression of ALD (Adachi et al., 1994). The activation of the inflammasome is triggered by a variety of potential molecules in ALD, including DAMPs and PAMPs. Alcohol-induced intestinal barrier, leading to gut permeability, increased along with the leakage of gut microbiota product lipopolysaccharide (LPS) (DeSantis et al., 2013). Subsequently, the LPS binds to the TLR4 on Kupffer cells, which acts as the priming signal to induce the inflammasome component’s gene expression, to activate the NLRP3 inflammasome (Ganz et al., 2011). Additionally, alcohol-induced hepatocyte damage results in the release of DAMPs (ATP and uric acid) and mediated inflammasome activation (Tilg et al., 2016). It was verified by clinical and experimental studies that the increase in ATP and uric acid have been found in alcohol-fed healthy humans (Petrasek et al., 2015) and mice that were fed the ethanol diet (Iracheta-Vellve et al., 2015). Both P2X7-deficient and uric acid-inhibited mice lack inflammasome activation in alcohol-fed groups (Petrasek et al., 2015).

Many kinds of TCMs that play an important role in the development of ALD and have been used for the treatment of ALD. *Lycium barbarum L.* (LB) [Solanaceae; Lycii Fructus], a traditional TCM has a wide range of pharmacological effects, such as anti-inflammation, antioxidation, and hepatoprotective, and is usually used for “nourishing the liver” in clinical Chinese medicine (Chang and So, 2008; Tang et al., 2017). Recently, the LB polysaccharides (LBPs), the liquid fraction extracted from LB, showed an effect on ameliorating the progression of ALD, *in vitro* experiments confirm that LBP could reduce ethanol-induced oxidative stress, apoptosis, and the underlying mechanism of this role is proved by inhibiting NLRP3 inflammasome activation (Cheng and Kong, 2011; Xiao et al., 2014a). Moreover, zeaxanthin dipalmitate (ZD), one of the carotenoids of LB, showed an inhibiting effect on the NLRP3 inflammasome via modulating P2X7 and adipoR1, and drastically reduced inflammation infiltration and accumulation of fatty droplets in the ALD model rat (Gao et al., 2019).

In addition, many other natural products from TCM are also able to improve the development of ALD, such as gentiopicroside and active terpenoids of *Gentiana Manchuria* Kitag*.* [Gentianaceae; Gentianae Radix Et Rhizoma] regulated P2X7-NLRP3 to decrease the accumulation of aminotransferases and triglycerides in serum and reduced liver lipogenesis (Li et al., 2018).

Quercetin, a flavonoid from many TCMs, and ginsenoside Rg1, a natural terpenoid derived from PG, both showed a marked decrease effect on serum AST and ALT production, ameliorating the liver histology by inhibiting the activation of NRLP3 inflammasome via blocking oxidant stress in alcohol-fed mice and rats (Liu et al., 2018; Yang et al., 2021).

### TCM for the treatment of liver fibrosis by inhibiting the NLRP3 inflammasome

Liver fibrosis is a result of chronic liver inflammation that is majorly regulated by the inflammasome, and the advanced form of fibrosis is responsible for liver failure (Bataller and Brenner, 2005). Numerous studies have indicated that the activation of the NLRP3 inflammasome is a critical contributor to the development of liver fibrosis. The inflammasome activator (uric acid crystals) increase the expression of transforming growth factor (TGF)-β1 to activate the hepatic stellate cells (HSCs), triggering collagen production and deposition in humans and mouse, but does not occur in ASC-deficient situation (Watanabe et al., 2009). Kupffer cell activation-dependent IL-1 also is an indirect factor in the progression of fibrosis, which activates HSC by binding to IL-1β receptors (Weiskirchen and Tacke, 2014). Furthermore, some experimental studies showed that NLRP3 or ASC deficiency protects mice from carbon tetrachloride (CCl_4_)-induced increase of hepatic TFG-β1 and collagen-1α1 expression. NLRP3 knock-out reduced liver fibrosis and inflammation in NASH model mice (Gaul et al., 2021).

25-0CH_3_-PDD (PDD), one of the ginsenosides derived from PG, showed an activation effect on LXRs to inhibit P2X7-mediated NLRP3 inflammasome activation, decreasing serum ALT/AST expression and ameliorating liver injury and fibrosis in thioacetamide (TAA)-induced mouse model (Han et al., 2018). The liver X receptors (LXRs) are considered a critical regulator of energy metabolism, which had been reported to downregulate inflammatory gene expression, including il-1β, il-6, P2X7, etc., inhibiting inflammation (Zhu et al., 2012). Recently, a study showed that ursolic acid, a natural terpenoid isolated from a variety of herbal medicine, decreased collagen deposition and fibrosis-related factors expression and inhibited the level of NADPH oxidase 4 (NOX4) and NLRP3 in the CCl_4_-induced liver fibrosis model. NOX4 activates liver fibrosis via regulating ROS to trigger apoptosis and HSC activation (Crosas-Molist and Fabregat, 2015). NLRP3 and NOX4 deficiency ameliorates the progression of ALD (Nie et al., 2021). In addition, alpinetin, a flavonoid isolated from *Alpinia katsumadai* [Zingiberaceae; Alpiniae Katsumadai Semen], also affects ameliorated liver injury and fibrosis via inhibiting NLRP3 inflammasome activation (Zhu et al., 2021b).

## Discussion

**FIGURE 2:**
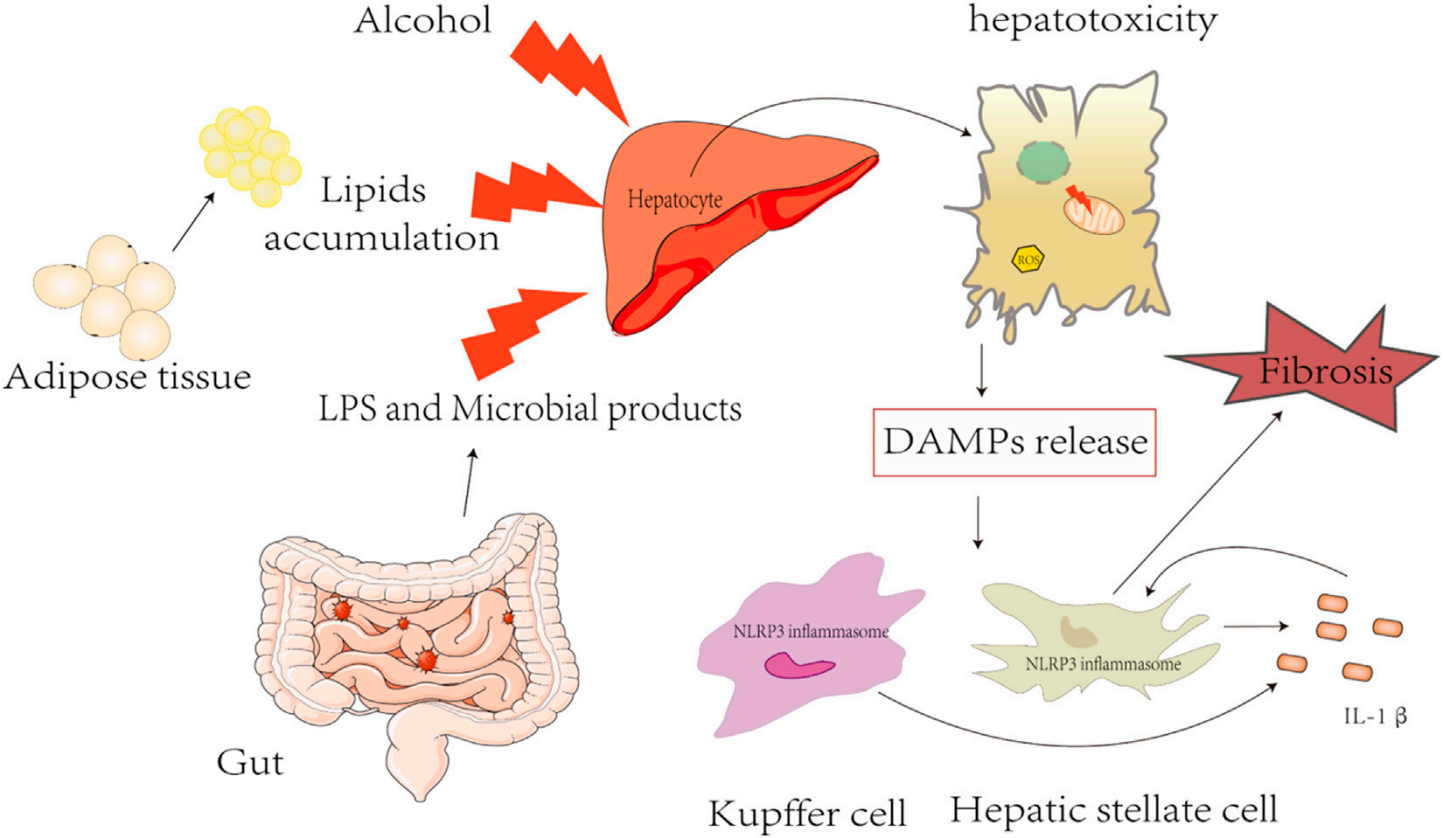
Triggers of inflammasome activation in fatty liver diseases.

NLRP3 inflammasomes play a pivotal role in FLD, especially in the progression of chronic types, including NAFLD, ALD, and liver fibrosis (Figure 2). However, the current knowledge of the mechanism of NLRP3 inflammasome activation is still very limited, and there is a lack of efficient clinical drugs for targeting NLRP3 inflammasome. Currently, therapeutic strategies are aimed at inhibiting the NRLP3 inflammasome signaling pathway by using NLRP3, IL-1β, TNF-α, and caspase inhibitors. MCC950 is well known as an NLRP3-specific inhibitor, showing a promising therapeutic effect in a variety of NLRP3-dependent immunopathological mouse models, including colitis, steatohepatitis, etc., but it was withdrawn from phase II clinical trial for the treatment of rheumatoid arthritis due to hepatotoxicity (Mangan et al., 2018). In addition, antibodies or antagonists (canakinumab) are used as inhibitors for IL-1β, which have been evaluated in humans (Kuemmerle-Deschner et al., 2011); however, multiple pro-inflammatory cytokines are induced by NLRP3 inflammasome activation and the treatment strategies to block IL-1β still need further study. Pentoxifylline has been known as a selective inhibitor of TNF-α and has been used in treating patients with severe alcoholic hepatitis in a randomized study, but it did not improve outcomes (Thursz et al., 2015). GS-9450 is an effective caspase inhibitor for caspases 1,8, and 9 and has been explored for NASH in a randomized, double-blind, placebo-controlled study, which demonstrated the potent effect in decreasing ALT levels safely and with tolerance. However, episodes of GS-9450-induced DILI occurred in a 6-month study in hepatitis C subjects, and the safety and efficacy of long-term caspase inhibitor in NASH still need to be further investigated (Ratziu et al., 2012).

TCM has been extensively applied for the prevention and treatment of various liver diseases, particularly FLD. Many Chinese herbal formulations, TCM extracts, and natural products exhibit beneficial effects on the progression of FLD via modulating the NLRP3 inflammasome pathway (Table 3, 4). Carnosol is one of the phenols isolated from *Rosmarinus officinalis* [Lamiaceae; *Rosmarinus officinalis L.*]; cryptotanshinone is a quinones components in *Salvia miltiorrhiza Bunge* and serves as therapeutics against NLRP3-drive disease, including LPS-induced mortality and MCD-fed induced NASH mouse model via inhibiting the activation of NLRP3 inflammasomes (Shi et al., 2020b; Liu et al., 2021). Therefore, TCM has shown promising therapeutic anti-inflammatory, antioxidant, and anti-fibrosis t that might take beneficial effects on curtailing the progression of ALD.

**TABLE 3:** Traditional Chinese medicine formulas for the treatment of FLD by inhibiting NLRP3 inflammasome activation.

Chinese medicine formulas	Common composition	Model	Effect	Mechanisms	Ref
Dansheng Zexie decoction	Baizhu, Zexie, Danshe (4:10:5)	HFD-fed rat model	Decrease lipid accumulation, alleviate hepatic steatosis and oxidative stress	ROS/NLRP3/IL-1β	Biao et al. (2022)
Shenling Baizhu powder	*Dolichos lablab, Poria, Glycyrrhiza, Platycodonis Radix, Nelumbinis Semen, PG, Amomi Fructus, Dioscoreae Rhizoma, Coicis Semen, Baizhu*; (4:5:3:2:3:5:2:5:3:5)	HFD-fed rat model	Reduce body weight, serum free fatty acid, and ameliorate liver microcirculation and ultrastructural abnormalities	TLR4/NLRP3	Pan et al. (2021)
Chaihu-Shugan-San decoction	*Citri Reticulatae Pericarpium, Bupleuri Radix, Chuangxiong Rhizoma, Cyperi Rhizoma, Aurantii Fructus, Paeoniae Radix Alba; Glycyrrhiza* (6:6:5:5:5:3)	HFD-fed rat model	Reduce serum LPS level, liver steatosis, and reconstruct the intestinal microflora	--	Yang et al. (2014)
Jinlida granules	PG, *Polygonati Rhizama*, *Atractylodes lancea*(*Thunb.*)*DC* [Asteraceae, *Atractylodis Rhizoma*], *Sophora flavescens Ait*. [Leguminosae, *Sophorae Flavescentis Radix*], *Ophiopogon japonicus* (*L.f*)*Ker-Gawl.* [Liliaceae, *Ophiopogonis Radix*], *Rehmannia glutinosa Libosch* [Scrophulariaceae; *Rehmanniae Radix*], *Polygonum multiflorum Thunb.* [Polygonaceae; *Polygoni Multiflorl Radix*], *Cornus officinalis Sieb. et Zucc.* [*Cornaceae, Corni Fructus*], Poria, *Eupatorium fortunei Turcz.* [Asteraceae; *Eupatorii Herba*], *Coptis chinensis Franch.* [Ranunculaceae; *Coptidis Rhizoma*], *Anemarrhena asphodeloides Bge*. [ Liliaceae; *Anemarrhenae Rhizoma*], *Epimedii Folium*, Danshen, *Lycii Fructus*, *Pueraria thomsonii Benth*. [Leguminosae, *Puerariae Theomsonii Radix*], *Litchi chinensis Sonn.* [ Sapindaceae; *Litchi Semen*] (10:12:6:5:12:9:8:12:8:5:5:6:5:8:8:12:12)	HFD-fed mice model	Alleviate insulin sensitivity and glucose tolerance, and suppress mRNA expression of caspase-1, IL-1β, and IL-18	Anti-pyroptosis	Hao et al. (2022)

**TABLE 4:** Botanical drugs and natural products for the treatment of FLD by inhibiting NLRP3 inflammasome activation.

Type	Botanical drug/natural product	Model	Effect	Targeted pathways	Ref
Extracts	Ethanol extracts from *Antrodia cinnamomea*	MCD-fed rat model	Attenuate steatohepatitis, oxidative stress, and hepatic inflammation	_	Yen et al. (2020)
	Water extracts from *Rheum palmatun L*	MCD-fed mice model	Improve serum inflammation and liver function	NLRP3-- ASC	Wu et al. (2022)
	*Lycium barbarum* Polysaccharide	MCD-fed mice model	Decrease serum ALT and AST levels, hepatic oxidative stress, fibrosis, inflammation, and apoptosis	NF-ΚB/NLRP3	Xiao et al. (2018)
		Ethanol-induced hepatocyte BRL-3A cells	Improve cellular apoptosis, inflammation, and oxidative stress	TXNIP-NLRP3	Xiao et al. (2014a)
Flavonoids	Licochalcone B	MCD-fed mice model	Decrease ALT and AST levels, liver inflammation, steatosis, and fibrosis	NLRP3-NEK7	Li et al. (2022)
	Echinatin	MCD-fed mice model	Decrease ALT and AST levels, liver inflammation, steatosis, and fibrosis	NLRP3-HSP90	Xu et al. (2021)
	Quercetin	Alcohol-fed mice model	Improve hepatic inflammation, reduce IL-1β, IL-6 expression and ROS release, and inhibit NF-κB activation	Heme oxygenase-1	Liu et al. (2018)
	Silybin	HFD-fed mice model	Reduce thioredoxin-interacting protein and IL-1β expression, caspase-1 cleavage	NAD^+^/SIRT	Zhang et al. (2018)
	Alpinetin	CCl_4_-induced mice model	Suppress liver inflammation and oxidative stress, decrease MDA level	NLRP3, Nrf2- mediate anti-oxidative stress	Zhu et al. (2021b)
	Baicalin	Free fatty acid-induced HepG2	Ameliorated morphological damage and death	NLRP3-GSDMD	Shi et al. (2020a)
Quinones	Emodin	MCD fed mice model	Improve serum ALT, AST, IL-1β, and TNF-α levels, hepatic inflammation, and fibrosis s	ASC oligomerization	Wu et al. (2022)
	Rhein	MCD-fed mice model	Improve serum ALT, AST, IL-1β, and TNF-α levels, hepatic inflammation, and fibrosis	ASC oligomerization	Wu et al. (2022)
	Diacerein	MCD-fed mice model	Improve serum ALT, AST, IL-1β, and TNF-α levels, hepatic inflammation, and fibrosis	ASC oligomerization	Wu et al. (2022)
	Aloe-emodin	MCD-fed mice model	Improve serum ALT, AST, IL-1β, and TNF-α levels, hepatic inflammation, and fibrosis	ASC oligomerization	Wu et al. (2022)
	Cryptotanshione	MCD-fed mice model	Decrease ALT and AST levels, improve hepatic inflammation, fat vacuoles, and fibrosis	Ca^2+^ signaling	Liu et al. (2021)
	1,8-dihydroxyanthraquinone	MCD-fed mice model	Improve serum ALT, AST, IL-1β, and TNF-α levels, hepatic inflammation, and fibrosis	ASC oligomerization	Wu et al. (2022)
Alkaloids	Berberine	MCD-fed mice model	Reduce mortality and ALT, TNF-α expression and phosphorylation of NF-κB	P2X7	Wang et al. (2021)
Terpenoids	Zeaxanthin Dipalmitate	LD-fed mice model	Improve hepatocyte autophagy, liver inflammation	P2X7 and adipoR1	Xiao et al. (2014b)
	Gardenoside	HFD-fed mice model	Improve ROS release, pyroptosis, and apoptosis	CTCF/DPP4	Shen et al. (2021)
	Gentiopicroside	LD-fed mice model	Decrease serum aminotransferases and triglyceride accumulation	P2x7R-NLRP3	Li et al. (2018)
	Glycyrrhizin	MCD-fed mice model	Alleviate serum bile acids accumulation, hepatic steatosis, inflammation, and fibrosis	FXR	Yan et al. (2018a)
	Ginsenoside Rg1	HFD-fed mice model	Reduce liver weight triglyceride, liver free fatty acids, MDA levels, serum ALT, AST, total bilirubin level, improve hepatic steatosis, hepatocellular apoptosis, mitochondria damage	—	Xu et al. (2018)
		Alcohol-fed mice model		Anti-oxidative stress	Yang et al. (2021)
	Ursolic acid	ETOH-fed mice model	Reduce lipogenesis and promote lipid oxidation	HMGB1-TLR4	Shang et al. (2022)
	Carnosol	MCD-fed mice model	Decrease serum aminotransferases, hepatic triglyceride, inflammation, and fibrosis	NLRP3-HSP90	Shi et al. (2020b)
	Carnosic acid	HFD-fed mice model	Improve glucose and insulin tolerance, decrease inflammation and lipid accumulation	PI3K/AKT, NLRP3/NF-ƘB	Song et al. (2018)
				SREBP-1C, MARCK	
	25-0CH3-PDD	TAA-induced mice model	Improve serum ALT, AST, hepatic transcripts of pro-fibrogenic markers, hepatocyte apoptosis	LXRs-P2X7R	Han et al. (2018)

In this review, we summarized and discussed recent research on Chinese herbal formulations, TCM extracts, and natural products that improve the status of ALD via inhibiting the NLRP3 inflammasome.

Most of them, such as *Glycyrrhiza*, and *Salvia miltiorrhiza Bunge*, are commonly used in clinical practice, and their formulations and natural products can achieve an anti-inflammatory effect inhibiting NLRP3 inflammasome activation, showing advantages in reducing side effects, improving prognosis during FLD treatment, and improving the survival rate of patients. Moreover, the natural products, such as licochalcone B and cryptotanshinone, have the potential to be developed as inhibitors for treating FLD due to their specifically inhibiting NLRP3 inflammasome activation. However, the mechanisms underlying TCM inhibiting NLRP3 inflammasome activation have not yet been systematically studied and remain to be further investigated. The bioactive components of TCM are complex and many of them exhibit an anti-inflammation effect, but it is unknown whether there is synergistic or antagonistic interaction between these components. Moreover, most research based on animals and cells is urgently required to undergo clinical safety and efficacy studies. Collectively, even though further studies are required to disentangle the mechanism of TCM, we believe that TCM and its natural products are promising therapeutic applications for the treatment of FLD.
